# From Querulous to Suicidal: Self-immolation in Public Places as a Symbolic Response to the Feeling of Injustice

**DOI:** 10.3389/fpsyg.2017.01901

**Published:** 2017-10-31

**Authors:** Benjamin T. Lévy, Cécile Prudent, Florian Liétard, Renaud Evrard

**Affiliations:** ^1^Interpsy Laboratory, Department of Psychology, University of Lorraine, Nancy, France; ^2^Loria Laboratory and Élie Cartan Institute, University of Lorraine, Nancy, France

**Keywords:** querulousness, self-immolation, suicidal thoughts, suicide attempt, psychoanalysis

## Abstract

**Aim:** This paper sheds light on the context that leads some querulous patients to self-immolate in front of, or into, public buildings (e.g., tribunals, city halls, and employment agencies).

**Method:** The author defines paranoid querulousness. A psychoanalytic perspective, but also a judicial and a psychiatric point of view, over querulous claimants is presented. The links between political or social claims and self-immolation are studied. The expression of suicidal thoughts voiced by four querulous subjects is analyzed. Eight examples of self-immolation are presented.

**Results:** The querulous subjects' self-aggressive behaviors seem to be caused by a loss of hope to obtain compensation for a prejudice they allegedly suffered. Querulous individuals tend to self-immolate in front of, or into, public buildings when no answer is given to their claims. These gestures may be both a consequence of some personal distress and triggered by a difficult social or professional context.

**Discussion:** Five sets of assumptions derived from Freudian and Lacanian psychoanalytic theories are advanced. The status of the object over which the querulous claimants wish to assert their rights is clarified. The meaning of self-aggressive gestures is outlined by making reference to the concepts of instinct for mastery, symbolic other, chain of signifiers, masochism, pleasure principle, and reality principle.

**Conclusion:** Prevention of self-immolation could involve that members of the legal professions, social workers, civil servants, and mental health professionals in contact with querulous subjects openly show their will to listen to these claimants' voice: self-aggressive gestures might be avoided by supporting the querulous person's hope to obtain compensation for the prejudice allegedly suffered.

## Introduction

Psychoanalytic contributions to forensic sciences have existed since the beginnings of psychoanalysis. They represent a field of research of their own. Freud insisted on the peculiar case of “criminals from the sense of guilt” (Freud, 1916); some years later, he devoted a few lines to the expert's opinion in the context of the Halsmann murder case (Freud, 1930). Ferenczi expressed his views on the links between psychoanalysis and criminology (Ferenczi, 1919, 1928). Lacan made a stand on the “paranoid crime” of the Papin sisters (Lacan, 1933) and his research would be continued by the “Theoretical Introduction to the Functions of Psychoanalysis in Criminology” (Lacan, 1950). Today, the nature of the relationship between these disciplines is questioned by a good number of papers published every year. Their authors aim at reconciling some psychiatric and criminological data with Freudian or Lacanian psychoanalysis (Assoun, 1997, 2004; Trichet and Hamon, 2016).

The main crossroad between psychoanalysis and criminology is represented by the topic of aggressive acts and/or behaviors. Jovelet (2006) has aimed at defining what can be qualified as an “act.” Millaud (2009) has shed light on the “clinical and psychodynamic” aspects of some aggressive behaviors. Other authors have focused on recurring modalities of aggressive gestures such as the “crime of passion” (Zagury, 2010), serial killings (Zagury, 2002), rapes (Bessoles, 2013), and the “unmotivated murders” committed by some schizophrenic patients (Adens, 2008).

In these pages, we suggest considering a kind of act which has been investigated by a surprisingly small number of psychoanalysts—namely the self-aggressive gesture usually called a suicide. It is not self-evident that gestures by which a man or a woman decides to put an end to his or her own life should be referred to as an “act” (Morel, 2004). And yet, suicide constitutes a case of lethal violence of its own right (Roh, 2017). We shall contribute to the debate on this subject-matter by describing the situation of some querulous individuals who have deliberately decided to put an end to their lives by self-immolating in public places so as to protest against an economic, social, and/or political injustice. Under what conditions may we advance that these subjects' self-aggressive tendencies shed light on the beginning of their life-story? Which are the missing links between an injustice suffered and a suicide attempt?

To help answering these difficult questions, we will first specify who the querulous subjects are, both from a psychiatric and from a judicial point of view. The characteristics of querulous individuals' suicidal behaviors will be described. This will contribute to determining what relates self-immolation in public places to some previous social, economic, and/or political injustice suffered. Clinical data will be presented to support our hypotheses: we will analyze how suicidal ideas were expressed by four querulous individuals whom we have interviewed twice. Then, examples will be given of suicide attempts in public places. In the last pages, we will discuss the possible meaning of self-aggressive gestures in a context of querulousness by making reference to the Freudian and Lacanian theories, and notably to the concepts of instinct for mastery, symbolic other, chain of signifiers, pleasure principle, and reality principle.

## Who are the “querulous” subjects?

### A psychiatric and judicial point of view

The notion of “delusional querulousness” was invented in Germany in 1857 by Casper (1857). Under the name of *Querulantenwahn* (claimant's delusion), he described the slow evolution of a disorder which led some subjects to relentlessly claim compensation for some damage they allegedly suffered. The *Querulant* (claimant) would first initiate one legal proceeding against one particular person considered as his/her enemy and then multiply his/her claims directed against an always larger group of individuals. Meanwhile, he/she would always oppose the decisions of the judges and accuse them of being unfair, biased, and utterly unable to uphold justice (Casper, 1857, pp. 543–544).

Although Casper didn't consider the *Querulantenwahn* an autonomous clinical entity, von Krafft-Ebing, in his textbooks, changed it into a subgroup of the persecutory delusion (von Krafft-Ebing, 1875, pp. 123–124). Kraepelin (1915) later separated these two disorders and, from the 1930s onwards, querulousness was considered as a subgroup of “psychopathic personality” by the German psychiatrists (Schneider, 1923).

In France, the same clinical data led to the birth to a new diagnostic concept, namely the *délires de revendication* (delusions of revendication). This expression was coined by Cullerre (1897). He described some “lunatics” driven by a passionate desire for justice, but who refused to comply with the judgments rendered by the courts. Thanks to Sérieux and Capgras (1909), the “delusion of revendication” became a subgroup of the paranoid delusions. De Clérambault (1921) would confirm that “querulousness” constituted a subtype of the “delusion of revendication.” He would insist on the pathological passion which, in his eyes, constituted the core of the delusional subject's character.

Today, the expression “quérulence processive” (litigious querulousness) may be pronounced by French psychiatrists when they meet a paranoid patient keen on suing his opponents (Fouldrin et al., 2006). Some German specialists may still speak of *Querulantenwahn* when they wish to refer to this kind of pathological behavior and/or personality (Tölle and Windgassen, 2011). But then, both terms only describe a mode of expression for paranoia. In other words, they refer to a mere symptom of severe mental disorder. This situation reflects the data indicated by the DSM-III-R to DSM-IV-R editions (American Psychiatric Association, 1987, 1994, 2000): in these works, the querulous paranoia is considered not a diagnostic concept of its own but a transient pathological phenomenon liable to appear during the course of any delusional disorder, persecutory type.

In English-speaking countries, querulousness has, in fact, never been considered a clinical phenomenon distinct from persecutory delusion (Soothill et al., 2013, p. 574). Historically, a judicial point of view on pathological claimants has prevailed over a psychiatric one. From the late nineteenth century onwards, in England and Wales, then in Scotland and in the whole of the former British Empire, from Australia to Canada, including India, Jamaica, and other countries, judicial measures were specifically adopted for banishing troublesome suitors from the courts by labeling them as “vexatious litigants” (Stauber, 2009). This expression is applied to citizens who have filed an excessive number of proceedings without any reasonable ground and have consequently lost their right to initiate new proceedings, except if a judge grants them formal leave to do so (Rowlands, 1988; Stauber, 2009).

Such measures exists neither in Germany—where a criminological debate exists over querulous person's dangerousness (Tölle and Windgassen, 2011; Knecht, 2012)—nor in France—where the responses which should be given to abuse of process remain a source of questioning for the judges (Kebir, 2016). This paper advances new elements which allow a better understanding of the problem. We shall focus on the querulous individuals' self-aggressive tendencies: at the moment when they become aware that they won't obtain satisfaction, suicidal thoughts, suicide attempts, and even effective suicide may prevail over their desire to live. What does this teach us about their querulousness?

### General features and context of the querulous person's suicide

As Casper noted as early as (Casper, 1857) (pp. 543–544), the querulous subjects first file legal proceedings against one person who is accused to have wronged them. Afterwards, their anger is directed toward the judges who are accused to have rendered an unfair verdict. They may also pretend that their lawyers have misled them and claim that police officers in charge of investigating the case have neglected to fulfill their task. Some of the querulous subjects whom we have met (*see below*) claimed that “justice had not been done.” One of them added that “the judges” had refused to “consider the elements of proof [he] had brought forward”. Unfortunately, these accusations may backfire and change into self-destructive behaviors. Bouazzoni (2013), Bourdais (2013), and Bollendorf and Collo (2013) advance that every 2 weeks, in France, one person attempts to self-immolates in front of, or into, a tribunal, a town hall, an employment agency, or another public place in order to protest against an injustice suffered. Psychiatrists all over the world have described similar acts which have been the object of detailed quantitative reporting (Laloë, 2004; Ben Khelil et al., 2016; Moradinazar et al., 2016).

The querulous subjects who contemplate self-immolating in public places invest their act with a strong symbolic value: it constitutes the continuation of a war they have been waging to promote a social, political, or professional cause (Belaid et al., 2012; Bette, 2013; Zaretsky, 2013). These individuals wish to “protest” (Bouazzoni, 2013) in an exemplary way. A claim, which has not been taken into account by clerks, judges, colleagues, or family members, leads them to use radical means. This phenomenon has deeply damaging social consequences. In Algeria, “the figure reported […] more than 100 victims [in] 3 years” (Belaid et al., 2012). In France, just like in Algeria or in Greece during the economic crisis (Bouazzoni, 2013), the media's discourse tends to spread the idea that these protesters try to use the impressive nature of their act as a means to support their claims. Meanwhile, journalists agree that self-aggressive behaviors may also be triggered by despair (Assouline, 2012). From a more scientific viewpoint, suicidal behaviors may be mistaken with the act which puts an end to a melancholic depression (Reniers et al., 2011; Lévy and Vanier, 2016). However, the querulous subjects' gesture seems to be less related with self-accusation (a pathognomonic feature of melancholia) than with a demand for social recognition.

In this regard, self-immolations which happen in public places may be endowed with a particular symbolic dimension. In the Western world, since the times of the Athenian democracy, public places have been a space devoted to the expression of political and personal beliefs. In ancient Athens, the Agora represented both a meeting place and a civic center which included the tribunal and the citizen's assembly (Orrieux and Schmitt-Pantel, 2013). In the cities of the Roman Empire, the *forum* used to play basically the same role (Watkin, 2009). Nowadays, simple citizens rarely have the opportunity to take part in political or judicial deliberations, and working may have become a more widespread means of social recognition (Renault, 2007; Didry, 2012). For those who feel deprived of this recognition and consider themselves as victims of injustice, committing suicide in front of—or into—a tribunal, a town hall, or an employment agency might be an effective way of expressing both their anger and their disarray. As we shall see below, carrying out their plans in public places may also allow them to captivate the public's and the media's attention, thus creating a shock effect. It may be an imaginary and partly unconscious way of inflicting on others some part of the harm which they have allegedly suffered.

### From political claims to self-immolation: many questions, few answers

Historically, self-immolation, protest and political claims have been correlated with one another. In 1969, Ian Palach self-immolated on Wenceslas Square in Prague in protest against the invasion of Czechoslovakia by the Red Army (Meddeb and Semelin, 2001); 20 years later, in memory of his sacrifice, some political opponents launched the Velvet Revolution (*ibid*.). In Tibet, those who self-immolate have become a symbol of the struggle against the Chinese occupation (Vernerey, 2012). In Tunisia and Algeria, similar acts characterize citizens who wish to make public their despair and/or support a political cause (Valter, 2011). At once a public act and an expression of personal distress, it may sometimes be difficult to know whether these self-immolations should be labeled as voluntary deaths or as suicides.

The latter two categories of self-inflicted death have been the object of an in-depth study by Kitanaka (2012). This author defines a voluntary death as the consequence of a conscious, rational decision. A suicide, by contrast, is supposedly caused by despair and depression. According to Kitanaka, suicide constitutes an expression of mental disorder: it testifies from the utter hopelessness and existential unrest of a singular person. Voluntary death, on the other hand, should be considered a rational means chosen by a purposeful individual to solve (or help solve) one given problem.

Another set of distinctions concerning suicide has been advanced by Baechler (1975). While recognizing that his categories are porous, he separates the “escapist” suicides (motivated by the wish to flee an unbearable situation), the “aggressive” suicides (whose objective is to impose harm on oneself and/or others), and the “oblative” suicides (which serve a person or a cause).

To help determine if the querulous subjects' self-aggressive gestures pertain to some of the latter categories, we will soon present the case of a few plaintiffs who have been drawn into a dynamics of social and political claims and who have voiced suicidal ideas. Their testimony let us clearly guess that potential suicides are less motivated by a rational decision than caused by despair. At the same time, an “escapist” dimension seems to prevail over an “oblative” dimension. However, let us not anticipate, for we still have to describe more in detail the context of these acts.

## Some hypotheses on suicidal behaviors

### General facts

Major depressive episodes have been discovered as an antecedent for 51% of suicidal gestures (Jehel, 2000) and psychiatric antecedents exist for up to 90% of young suicide candidates (Righini et al., 2005). Berman (2017) indicates that most suicide attempts have, in themselves, underlying psychiatric causes. For schizophrenic patients, suicide rarely happens during the course of psychotic episodes, though suicidal ideation exists in 60–80% of cases (Jehel, 2000). Divorce and a context of separation, mourning, and widowhood are positively associated with suicide (Lester, 1993; Gibbs, 2000); however, there is no proof of a direct causation. Some studies established a relationship between self-aggressive behaviors and some hardships encountered in professional life but, here again, the existence of a direct impact of unemployment on suicide rates remains controversial (Charlton, 1995; Noh, 2009; Iglesias-García et al., 2017). One study which has been carried in Algiers, Algeria, has identified 92 cases of self-immolation in public places in 25 years (1987–2012) (Belaid et al., 2012). The authors observed that self-immolation mostly affects young people (mean age 28) of the male sex (73%). Belaid and colleagues advance the idea that they suffer from an “adjustment disorder” which doesn't constitute a mental disorder of its own but which may accentuate a thorough emotional distress already exacerbated by stressing events (family problems, exclusion, and deprivation). The same study underlines that immolation, when committed in public, represents a sacrificial action. Its careful execution may hint at a will to take revenge over some relatives, to blame them and, in the same time, to arouse emotion.

Other sources indicate that, in the western world, self-immolation accounts for 0.7–14% of all patients admitted in burns units (Garcia-Sanchez et al., 1994; Laloë, 2004; Moniz et al., 2011). These acts, which are not always carried out in public places (Nakae et al., 2003; Makhlouf et al., 2011), shouldn't be mistaken with voluntary scarifications by burning (Sonneborn and Vanstraelen, 1992; Balakrishnan et al., 2007). Meanwhile, they represent 0.75–2.8% of all suicide attempts (Shkrum and Johnston, 1992; Rothschild et al., 2001; Ahmadi and Ytterstad, 2007; Moniz et al., 2011). There is a strong prevalence of men among people who self-immolate (Garcia-Sanchez et al., 1994; Rothschild et al., 2001; Gauthier et al., 2014). Their mean age is situated between 28 and 43 (Shkrum and Johnston, 1992; Rothschild et al., 2001; Poeschla et al., 2011). Mortality rates vary from 9 to 89% (Laloë, 2004). Mean total body surface area (TBSA) of burns is significantly higher for men than for women (37.2 ± 27.7% vs. 23.2 ± 16.6%; *p* < 0.05) (Moniz et al., 2011, p. 324). The method of self-immolation most employed is pouring highly inflammable liquids (benzine, petroleum, alcohol, and kerosene) on the clothes and setting fire to them (Hadjiiski and Todorov, 1996; Makhlouf et al., 2011).

Our own study—which has been carried out within the context of doctoral researches in clinical psychology based at the Paris Diderot University (Lévy, 2016a,b)—allowed us to meet and dialogue with 18 subjects engaged in long-term litigation after they had suffered a prejudice. Four of them (22%) mentioned suicidal ideas; however, they didn't commit a suicide attempt. The data presented in the following pages are extracted from these doctoral researches. They were gathered with the written consent of all participants, whom we interviewed twice. They were informed about the objectives and the procedure of the survey. Ethical criteria were met in accordance with the Helsinki declaration and the national French guidelines for psychological research. No formal approval by the ethics committee of the Paris Diderot University was needed, as our study didn't fall under the law on bioethics.

In these pages, all names and personal details have been changed. First, we will present how suicidal thoughts were voiced by the plaintiffs whom we have interviewed. Then, we will briefly show that aggressiveness which couldn't be directed toward others people reversed into self-aggressiveness. Thirdly, we will give examples of suicide by self-immolation in public places and, as far as it is possible, indicate their context.

### How do querulous claimants voice their suicidal thoughts?

The following clinical cases have been studied within the context of the above mentioned doctoral researches in clinical psychology written by the first author of this paper (Lévy, 2016a,b). The main criterion for selecting and presenting them is that they illustrate the multiplicity of personal situations which may lead a querulous subject to express suicidal tendencies. However, the following pages also enable to single out common points between plaintiffs who have suffered a prejudice and filed legal proceedings in vain. As we shall see below, this situation may lead to effective suicide by self-immolation.

#### M. Maubert: evoking an altruistic suicide

M. Maubert has been swindled for more than 30,000 euros. He advances that health problems caused a moment of weakness: “I was very weak,” he keeps repeating. He tried to invoke justice in vain on several occasions. Later, at his children's request, he was called to “appear before a court” [his expression] in order to be “placed under guardianship.” When we met him, he saw himself as a burden for others. The description of his utter loneliness led him to voice suicidal thoughts. His death would benefit his children, he said.

Similar self-aggressive acts motivated by a mixed sense of guilt and duty toward others were called by Emile Durkheim “altruistic suicides” (Durkheim, 1897, p. 233). Baechler (1975) would underline the presence of both an “oblative” and an “escapist” dimension in the suicidal ideas. For M. Maubert, the sudden loss of an important amount of money seems to have revealed a void, a nothingness which had, until then, remained hidden from his eyes.

This case (fully described in Lévy, 2016b, pp. 397–402) seems to constitute an example of some intermediate position between a querulous and a melancholic stance. After several unsuccessful attempts, M. Maubert realized that his legal recourses wouldn't meet with success. He fell into a melancholic depression. Some melancholic patients may try and kill their family before turning their weapon against themselves. This attitude may be motivated by the will to spare their children and wife what they consider an unbearable existence (Morali and Baratta, 2011). While this seems unlikely to happen here, M. Maubert's case teaches us that suicidal thoughts may appear in an intermediate phase, when the loss of hope to regain the object lost prevails over the wish to defend one's right by appealing for justice.

#### Ms Zuliani: querulousness as an hysteriform reaction to the loss suffered

Ms Zuliani's claims sound more hysteriform than melancholic. Around 50 years old, she has “lodged several complaints” against a “very clever” man. While having an affair with him, she trusted him with an important sum of money which, she thought, would help create a humanitarian association. The man disappeared with the money. Ms Zuliani considers that she has been “financially and personally humiliated” by these events. Her unfortunate experience “had a strong physical impact on her”: she has “lost 20 kilos” and, since then, she feels unable to trust other people.

When asked what she contemplates doing, Ms Zuliani reacts strongly. She is determined to “fight” until “truth is established.” The metaphor of a “struggle” or “fight” keeps coming out over and over in her speech. She asserts her will to “fight until death” if necessary. A sacrificial dimension punctuates her answers: in her eyes, the triumph of the truth seems to be worth the offering of her life. At the end of our interview, Ms Zuliani asks: “What should I do? Commit suicide?” but she immediately rejects the idea: “It's a solution, but I won't do it.” The evocation of suicide and the melancholic mood which accompanies it are negated. The well-known assertion re-emerges: “I'm a fighter. I live for my daughter.” Ms Zuliani concludes our interview with these words: “At first, I didn't understand what was happening to me. But now, I won't give up.”

Ms Zuliani's stance (fully described in Lévy, 2016b, pp. 461–464) as well as her somatic complaints seems to indicate a hysteriform reaction to the prejudice suffered. A sense of shame may have been aroused in her by the loss of an important amount of money, but this shame has been displaced. It now bears on the melancholic mood caused by the loss. When suicidal ideas emerge into her consciousness, they appear to be repressed. The self-sacrificial dimension of her struggle is denied, sometimes openly, sometimes more subtly. Her identification with the image of a “fighter” may enable her not to break down. Still, we don't know what would happen if no court judgment was issued in her favor. Would she be able to move on?

#### M. Gac: the exemplarity of martyrdom in a context of paranoid querulousness

M. Gac's case (fully described in Lévy, 2016b, pp. 69–77) is interesting. He may be the only genuine querulous-litigious plaintiff who has agreed to be interviewed by us. His speech and actions bear the distinctive signs of the disorder which is called in some French classifications a “delusion of revendication” (Fouldrin et al., 2006). In the English-speaking world, psychiatrists would rather talk of a “querulous paranoia” (American Psychiatric Association, 1987, 1994, 2000).

M. Gac advances that he has been cheated out of his inheritance. A farm which belonged to his father was inherited not only by him but also by his stepsister and the latter's husband. As a child of his father's first wife, M. Gac considers himself a “victim of [his parents'] divorce.” He has filed numerous proceedings to assert his rights over the domain, a strategy which didn't prove successful. So, he began two hunger strikes in vain. Since then, he has constantly been trying to attract the journalists' attention by walking across France, from his home town to Paris, stopping in every village to publicize his plight.

When interviewed, M. Gac declared that he had been separated from his father by a possessive mother. He enumerated the “sanctions and discriminations inflicted” to other children who, just like him, had been a “victim of [their parent's] divorce.” He underlined that, among them, “much higher rates of suicide” prevailed. He stated that his situation was “not uncommon” and endowed suicide attempts with an exemplary statute. On the one hand, he denied any personal wish to commit suicide; he presented himself as a “warrior” determined to fight an “enemy” who had “no feelings.” But on the other hand, the description of his unsuccessful appeals to justice led him to hint at suicidal ideas: “I've also thought about it. I had in mind to do it in front of the opposing party's lawyer's working place.” He described the suicidal thoughts which “sprouted” into his mind as a by-product of his opponents' will: “They push us into suicide, you shouldn't forget that.” However, rather than putting an end to his life, he went on struggling by addressing the media and raising public awareness for his cause.

For M. Gac, suicide seems to retain an exemplary statute: self-immolation equates to taking justice into one's own hands. Those who commit this act deserve a posthumous triumph.

#### M. Doux: a paraphrenic litigiousness?

M. Doux appears to suffer from a disorder often mistaken with schizophrenia, namely, paraphrenia (Lévy, 2016a, pp.246–254). This term was used by Kraepelin; (1915, pp. 973–1022) from the 8th edition of his *Textbook of Psychiatry* onwards. It refers to a form of psychosis situated between paranoia and schizophrenia. This disorder is characterized by “the apparent contradiction between absurd, abundant delusional ideas and a seemingly good adaptation of the subject to the constraints of reality” (Hulak, 2008, p. 12).

After our first encounter, M. Doux sent us documents that attested to his extraordinary beliefs. He showed no proof of symptoms which would have enabled us to diagnose schizophrenia (World Health Organization, 2016). However, he had filed dozens of complaints and expressed peculiar beliefs about UFOs, Mithraism, Freemasons, Rosicrucians, and others. The manager of a small business created by him had changed the legal statutes of his firm without anyone else's consent. This event and its consequences had dramatically altered his life, which led him to voice suicidal thoughts. In our views, the development of an exuberant conspiracy theory enabled him to overcome his suicidal ideas. A paranoid delusion accompanied by bizarre beliefs appeared along with the blossoming of an overflowing imaginary activity typical of paraphrenia. Suicidal thoughts re-emerged after every single defeat or misfortune met in the real life. Death constituted an always open “alternative solution” for M. Doux. He underlined that most victims of “dispossession […] end up with a severe chronic illness, unemployed, isolated, ruined, divorced […], or they commit suicide.” In M. Doux's statements, the suicidal idea was often evoked and then revoked. He switched to other topics. The frightful loss and the even more frightful suicidal reaction were covered by delusional representations.

This last example (fully described in Lévy, 2016b, pp. 89–99) allows to underline the diversity of personal characteristics and situations presented by querulous plaintiffs. The spectrum of querulousness spans from melancholia to paraphrenia; it includes paranoid delusions and hysteriform reactions. Further research would be needed to determine when querulousness becomes a pathological behavior, as opposed to a simple personality trait. It would also be important to study more in depth the context in which suicidal ideas such as those which have just been described turn into action and give way to suicide attempts. To begin shedding light on this matter, we shall now present 8 examples of actual self-immolations that have been carried out in public places.

### Suicide by self-immolation in public places. 8 Examples

In France, one suicide attempt by self-immolation in a public place occurs every 2 weeks (Bollendorf and Collo, 2013; Bouazzoni, 2013; Bourdais, 2013). No psychiatric or medical study has yet investigated the question and, in the course of our own research, we have not met any querulous person who had previously attempted suicide. We encourage further research. To pave the way for future studies, we have randomly selected a non-exhaustive list of newspaper clippings that describe suicidal gestures committed by querulous persons. The main criterion for our choice was that the general causes for the suicide attempt, which took place in public places, were known. These gestures were committed by seven French and one Ivorian citizen. The examples cover a 9-years period (2008–2017). They tend to prove that common points exist, both in the events which triggered the suicide attempt and in the way it was carried out. Nevertheless, the methodology employed has some limitation; most notably, we regret our inability to give information about the medical and/or psychiatric history of the subjects involved.

– In February 2008, a mother of two self-immolated in public, in a French city. A real estate agent had ruined her. She considered herself a victim of this man's incompetence and disloyalty. She also accused her lawyer and several credit institutions. Accumulation of debts and failure to win her case reinforced her wish to put an end to her own life (Delompré, 2008; Lesourd, 2008).
– In March 2009, a man in his thirties ignited his garments soaked with gasoline inside another French city's high court. He was quoted as accusing his country's justice of being unfair and inefficient while running through the building. This man wasn't summoned to appear in court on that day. According to the police, he had already expressed recurring grievances against the judges for economic losses he had suffered. They hadn't been compensated for (Le Parisien, 2009; Tian, 2009).
– In February 2013, an unemployed middle-aged man no longer eligible for social benefits put an end to his life by self-immolating in front of his employment center. Civil servants and journalists had received messages announcing his intention to act in protest against the rejection of his appeals. Measures adopted proved unable to prevent the gesture (AFP/Libération, 2013).
– In the same month, an unemployed citizen attempted to self-immolate close to a primary school. This man explained his gesture by alluding to his personal situation. He wasn't eligible for social benefits any more. A background of family problems existed. He had been heard complaining about his loneliness (RTL, 2013).
– In May 2013, a woman claiming a “right” to housing poured petrol over her head in the town hall of a small French town. She had previously asked to see the mayor with the aim of supporting her application for housing. Police officers managed to deter her from self-immolating (Paris-Normandie, 2013).
– In April 2014, a former police officer in his forties self-immolated in front of the tribunal of a small French town. Accused of theft – wrongly, he said – he had been dismissed. He acted in protest against what he considered a false accusation which had turned him into an unemployed citizen (Petitlaurent, 2014).
– In April 2016, a small business entrepreneur self-immolated at his workplace. The business he ran had gotten into severe difficulties and this man was unable to cope with the situation. He had applied for a financial loan but he could not accept the risk of going bankrupt (Le Monde/AFP, 2016).
– Far from the French borders, the same kind of gesture is described in Ivory Coast. To claim her due, a woman self-immolated in May 2014 in front of the presidential palace. “I want to die. They stole my life,” she was quoted as saying. A background of political militantism and serious dissatisfaction with the government policies existed (LS/APA, 2014).

These eight suicide attempts were carried out in public places. They may have been meant as ways of arousing the media's attention on a particular social, legal, or political issue. More in-depth studies should be encouraged to improve our understanding of the personal motivations of those who committed such acts. In particular, psychiatric history of the people who self-immolated should be investigated. This may help prevent other suicidal gestures (Eagles et al., 2001; Matakas and Rohrbach, 2007). In order to sum up our clinical data and to introduce our discussion, we shall now underline two important points.

### Killing oneself or killing the other?

In all 4 cases described within the section How do Querulous Claimants Voice Their Suicidal Thoughts? of this paper, the subjects declared that they had suffered prejudice. They had filed legal complaints to obtain redress. The tribunals represented a third party between themselves and their opponents. Unfortunately, legal proceedings remained unsuccessful. Aggressive feelings which couldn't be directed toward their adversaries seem to have turned against themselves, causing suicidal thoughts to appear. In a few pages, we will advance that the consequences of such a process may be described in terms of masochism; but for now, let us present some clinical traces of this phenomenon:

– Ms Zuliani acknowledged threatening her antagonist: “I told him, that's true, I told him: ‘I’ll kill you, I'll kill you.”' However, this statement was followed by apologizing for her own outburst and resuming a self-sacrificial fight for her legal rights.
– M. Gac engaged in a legal battle against his step-sister and the latter's husband. Meanwhile, he gravely endangered himself through hunger strikes.
– M. Maubert plunged into a melancholic depression after having been defrauded. He contemplated suicide both as a means to alleviate his own pains and as a way to help his children get rid of himself.
– M. Doux embodied the character of a hapless victim who had no choice but to remain suspended to his delusional ideas or to resort to suicide as a last “solution.”

Further efforts would be needed to explore the shift from self-sacrificial behaviors to suicide attempts. This would enable to discover if there exists a continuum between the plaintiff's feeling of injustice, their self-aggressive tendencies and their possible self-immolation. Meanwhile, describing the common features of suicide attempts committed in public places by querulous subjects may help us better understand the meaning of these gestures.

### Suicide by self-immolation. common features

None of the people interviewed in section How do Querulous Claimants Voice Their Suicidal Thoughts? had attempted suicide. However, the complementary data presented in section Suicide by Self-Immolation in Public Places. 8 Examples and a brief survey of the literature related to the topic may allow to describe in general terms the modus operandi of those who wished to put an end to their life.

– According to all sources, the first common feature of the querulous subjects' self-immolation is a public dimension (Assouline, 2012; Bouazzoni, 2013; Zaretsky, 2013). These gestures are committed in front of buildings endowed with some symbolic value (town hall, tribunal, and employment agency). The querulous plaintiffs carry out their suicidal project before the eyes of those who didn't want to grant them what they yearned for. As we shall see below, some masochistic and, on another level, some sadistic trends may be at work.
– The causes of these acts are rationalized. Personal problems seem to be denied. The suicidal gesture must be made public so as to become an accusation. The querulous individuals tend to accuse their society as a whole. The others, they say, pushed them into suicide (Bette, 2013).
– As a consequence, the querulous plaintiffs behave like martyrs. They take it upon themselves to cleanse away their society's most “burning” problems. They may describe themselves as exemplary citizens who struggle against injustice or put forward their altruistic intentions. Their sacrifice is supposedly an exemplary manner to improve the social functioning (Bouazzoni, 2013). These people may take it upon themselves to suppress what the psychiatrists Constantin von Monakow (1919) then Paul Guiraud (1931) called the “*kakon”*, an approximate equivalent of Melanie Klein's “bad object” (Klein, 1935).

In order to back up our hypotheses, it remains important to carry out some additional research which may give us a better insight into the medical and/or psychiatric past of those who committed self-immolation in public places. For now, we shall proceed with the discussion of our clinical findings.

## Discussion

Basing on the data presented, we will advance five sets of assumptions derived from Freudian and Lacanian theories. Our clinical findings will be worked through a variety of lenses. This will enable us to underline what seems to be at stake in the querulous subjects' claims. It will also lead us to single out some unconscious factors which may induce self-immolation.

### Instinct for mastery in querulous behaviors

To begin this discussion about self-aggressive acts associated with querulous behaviors, let us first present the notion of “instinct for mastery” (“*Bemächtigungstrieb”* is the original German expression) (Laplanche and Pontalis, 1968, pp. 364–367). It made its appearance in some of Freud's texts; however, “its use can't be precisely codified” (*ibid*., p. 364, my translation), which makes it difficult to understand.

In German, the verb “*sich bemächtigen”* means “to appropriate” something. It evokes the idea of domination by sheer strength. The expression “*Bemächtigungstrieb*” remains difficult to translate: some French authors have used the expression “*pulsion de maîtrise*” (mastery drive) or “*instinct de possession”* (instinct for possession) (*ibid*., p. 364). In spite of such lexical variations, cross-checking the occurrences of this term in Freud's text allows to discover its meaning. First of all, the “*Bemächtigungstrieb*” is an active tendency for appropriation, if need be at the expense of others. This tendency is illustrated by the child's gesture when he reaches the hand to grasp his mother's breast. When he makes such a move, the child doesn't wonder whether his siblings, be they younger or elder, may assert a claim to take his or her place. In the *Three Essays*, Freud advances: “Cruelty in general comes easily to the childish nature, since the obstacle that brings the instinct for mastery to a halt at another person's pain—namely a capacity for pity—is developed relatively late.” (Freud, 1905, pp. 192–193) The instinct for mastery makes its appearance before the development of the child's ability to sympathize with other people's sufferings. In other words, mastery is an exclusive right.

In Freud's views, the instinct for mastery contributed to the fulfillment of the child's tendency to action, a phenomenon closely linked with the development of his muscular strength (Freud, 1905, p. 196). Directed toward an object, this instinct supposedly reversed the subject's passive position into an active position (*ibid*.). It functioned as a means to satisfy some desire for revenge. Interestingly, the word “revendication” derives from the Latin verb “*vindicare*” (to avenge [oneself]), which takes us closer to this paper's topic.

Freud gave up theorizing about the instinct for mastery partly because he discovered that a desire for mastery underscored every manifestation of the *libido* through instinctual impulses (Sédat, 2009). He concluded that the instinct for mastery didn't represent an entity of its own: it rather constituted a prototype for the drive theory. Allied with sexual components, mastery could, Freud said, be changed into sadism (Freud, 1913, p. 322). This phenomenon is reflected by the querulous plaintiffs' insistence on seeking justice and asserting their rights even at the expense of others. Although Freud abandoned it, the notion of “instinct for mastery” offers a theoretical model that allows to better understand how delusional claims appear and multiply. Querulous behaviors seem to imply some tendency for mastery over what is demanded.

In Lacanian terms, this might mean that the object sought by the querulous individuals occupies the place reserved for the “objet petit a” (small a object) (Lacan, 1956–1957; Vanier, 2009). The latter embodies a fantasy-object which supports the subject's desire (Lacan, 1962–1963, p. 119), thus giving a target for the drive. At first, Lacan identified the “small a object” with the toy used by some children in the game of *fort-da* (Vanier, 2009, p. 39); but later, he accentuated its relation to the other. According to the Lacanian theory, the small a object is a residual part—a remainder—of the other (Lacan, 1962–1963, p. 135). We will see that, for querulous plaintiffs, the inability to get access to this object or to send a message to the other may have dramatic consequences.

### A failure to obtain the object and its consequences

In the querulous people's life story, there comes a time when some institutional figure refuses to grant the subjects what they yearn for. This fatal twist is epitomized when tribunals don't render a “satisfying” verdict. It may also be that employment agencies don't provide the claimants with a steady income or that social services don't grant them accommodation, etc. At some point, the claimants' target seems to vanish into thin air because some bearer of the symbolic order barred the way which supposedly led to it. According to the Lacanian theory, we may talk of some contingent deprivation that echoes symbolic “castration” (Lacan, 1956–1957, p. 269). Unfortunately, this event proves devastating. The claimant collapses.

Why does this reaction take place? Our hypothesis is that querulous individuals are convinced that *behind* the object they are aiming at, there is nothing. The sought-for object seems to be their last chance. It all happens as if the chain of signifiers abruptly ended there.

After it, there is a void. The subject cannot fill this void with a new signification that would support both his demand for love, which is situated beyond the discourse, and his desire, which lies underneath (Lacan, 1960–1961, p. 254). So, when a figure endowed with some symbolic authority (be it a judge, a deputy mayor or a civil servant, etc.) announces to these plaintiffs that they won't get what they yearn for, some melancholic reaction can draw near and a self-aggressive act may happen.

Here, we begin to better understand that the querulous claimants relate, at the same time, to the object considered as a target and to the figure which symbolically bars the way to it. In the querulous persons' eyes, this symbolic figure seems to be endowed with some paternal dimension. It deserves some respect. Self-aggressive acts stem from a failure to vanquish the father and conquer the object. In turn, the object—be it a sum of money, an accommodation, or a job—appears to be endowed with a maternal dimension. Just like Melanie Klein's “good” breast (Klein, 1957), which is a maternal attribute, it is perceived as a source of comfort and a provider of life. A mock-œdipian situation may emerge when the symbolic authority (paternal dimension) refuses to grant the (maternal) object. This pattern repeats an early triangulation.

If our assumptions prove right, then it would imply that some querulous subjects attempt to take their life at the very moment when they feel neither able to suppress the paternal figure nor to give up their quest for the maternal object. In a context of psychosis, this situation is amplified by a distortion in the realm of identifications (Lacan, 1958, p. 571). The other who refuses to give what is demanded may be compared to Schreber's almighty and terrifying God (Freud, 1911; Lacan, 1955–1956). His presence is felt both as a threat and as a source of excessive, inhuman bliss (Lacan, 1958, p. 569).

In this context, death seems to represent an escape from the other's grasp and a way of getting closer to the object sought. The querulous people who attempt suicide may wish to suppress the other's figure and also to reach in a hazy afterworld what they didn't possess when alive. Their self-aggressive gestures seemingly enable them (in their own fantasy world) to get access to their target while achieving the final aim of the pleasure principle: through suicide, they drastically and eternally lower their level of “internal tension due to stimuli” (Freud, 1920a, pp. 55–56). Let us conclude that their act represents both a response to an intolerable situation and an imaginary way to complete their quest.

### An act in-between the pleasure principle and the reality principle

As we have seen, the querulous claimants' self-destructive gestures seem to take place when all access to the object sought has been barred by a symbolic other, which bears no human face. At some point, the latter may play the role of an arbitrator, but when the legal battle turns into a dead end, a new cycle can't begin and aggressive feelings turn against the plaintiff himself. Emile Durkheim, the French sociologist, noted that the quest for an unreachable object, when accompanied by dissatisfaction with one's own situation, might be a cause of suicide (Durkheim, 1897, pp. 272–282). When the querulous plaintiffs' desire meets nothing but the harsh reality, and when no new hope draws their attention, death may appear as a solution. We shall try and reconcile this data with two theoretical points, the first of which pertains to sociology and the second to psychoanalysis.

From a sociological perspective, a study by Chua (2014) was devoted to the causes of suicide in the South Indian state of Kerala, which saw an accelerated growth and became one of the highest in India. In her book, the author endeavored to discover why suicide rates have soared along with the economic development. She concluded that members of a society who gets richer become more focused on consumerism. Then, suicide may change into a more common action which symbolizes the failure of individualistic ambitions. Citizens of modern, democratic nation are supposedly programmed to seek objects that satisfy their desire; inability to keep up in the race for material wealth and consumerism may be experienced by some vulnerable people as a fatal disgrace.

Closer to clinical data, Freud noted (Freud, 1920b, pp. 147–148) that one of his patients, a young female homosexual, committed an “undoubtedly serious attempt at suicide” just after she met her father and read in his eyes a note of disapproval at her behavior. This young woman used to walk arm in arm with another older woman (maternal dimension) she had fallen in love with. The first phase of the event is endowed with a theatrical dimension which is shattered by the father's anger. His disapproving eyes may have signified to the daughter: “You haven't any right to touch this maternal object.” The episode leads to a suicide attempt as the young woman jumps off from a bridge. Lacan commented on the German verbs “*niederfallen”* and “*niederkommen”* which both refer to parturition, downfall and degradation (Lacan, 1962–1963, p. 130 and p. 196). The whole story took place under the father's gaze, from its beginning (theatrical behavior) to its tragic ending (departure from the scene). This may provide us with a theoretical model for self-aggressive acts: they are a consequence of some abrupt transition from the pleasure principle (theatrical quest for the object) to the reality principle (interdiction or impossibility to carry out the fantasy). In other words, a subject may attempt to take his or her own life when he or she can't compensate for a real loss through the creation of a new fantasy.

### Suicide as the exhaustion of a chain of signifiers

According to Miller (1999, p. 5), Lacan's second paradigm of jouissance shows the existence of an equivalence between the fantasy and the chain of signifiers. In Seminary 17, Lacan gave a close look at this question: he defined the discourse as “a necessary structure which goes far beyond the simple speech” (Lacan, 1969–1970, p. 11, my translation). Indeed, the subject needs to articulate a discourse in order to support his fantasy. The latter, in turn, encloses his desire. As a consequence, the exhaustion of the subject's discourse may equate to a disorganization of his own fantasy world. This contributes to explain why the querulous plaintiffs may attempt to take their own life when they feel that they have lost all the chances to get access to the object through the use of language. Silence follows their noisy requests. Up to this point, the plaintiffs had been trying to pay off their debts to the others by using words. Their use of language allowed them to express their demands and to support their unconscious fantasy. In this respect, their behavior was similar to that of any other person. However, events unfold as if the other's negative response forbade them from sending their message. Then, they fall beyond the mirror of language and receive their own (reversed) message as a demand from the other.

Considered from this perspective, the decision to self-immolate constitutes a means to satisfy the other's (imaginary) demand. To do so, the querulous subjects may have nothing else to offer than their life. Since they can neither take their distance from the object sought nor support their desire by a newly created fantasy, they adopt a scorched earth policy and suppress themselves. In the same time, the choice to self-immolate may hint at a will to purify themselves from their incestuous love for the object sought (Bachelard, 1938). Their burning love for the object appears to be superseded by a burning message which, in turn, generates enjoyment effects. The latter resemble the Danaides' barrel (Lacan, 1969–1970, p. 17). They are based on an unfillable void. In seminar 17, Lacan presented an equivalence between the *jouissance* created by the radical absence of any object and masochism. He described the whole process as a road to death, adding: “Once you have started, you never know where it will end. It begins with a tickle and ends in a blaze of petrol.” (*ibid*.).

Whereas the depressive phase of mourning usually allows the non-querulous individuals to sacrifice some part of the object and its enjoyment (Freud, 1917; Allouch, 1995), suicide seems to reflect a failure of mourning and the subsequent impossibility to take some distance from the object, even when the latter is already absent. A close reading of Freud's text *Mourning and melancholia* (Freud, 1917) enables to understand this process. However, we have shown in a previous paper (Lévy and Vanier, 2016, p. 170) that the melancholic phases which may appear during the course of a (paranoid) querulous-litigious delusion should not be mistaken with genuine melancholy, which constitutes an autonomous clinical entity. One differential criterion is that, even at the worst of times, the querulous-litigious plaintiffs keep accusing their opponents (or the judges, the police officers, the civil servants, etc.) more than they accuse themselves. By contrast, self-accusations are a pathognomonic feature of genuine melancholy; psychoanalytical interpretation is needed to reveal that they hide accusations against others (Freud, 1917, pp. 245–248).

Between genuine melancholy and the depressive (“melancholic”) phases that appear during a querulous-litigious delusion, there is no equivalence. There are only analogies. For instance, in both cases, some enjoyment effects are created by accusations (or self-accusations). The latter generate sacrificial (or self-sacrificial) behaviors. To show the implications of this point, let us look back on a famous situation described by Freud (1900, p. 509), namely the account of a dream dreamt by a father who had lost his son. As the child's body was lying in the room next door under a guardian's responsibility, the father fell asleep. He dreamt that his son was calling and accusing him: “Father, don't you see I'm burning?” The father woke up. He became aware that the guardian was asleep. His son's body had begun being burnt by a candle. On a metaphorical level, we may wonder whether the partial destruction of the son's body within the dream constituted an image by which the father unconsciously attempted at symbolizing his own denial over the very existence of a son. The father's *unconscious demand* seemed to be here as a demand of filial sacrifice. For some querulous individuals, could self-immolation be a means to satisfy such a demand?

An observation may help answer this question. In countries which have adopted a special legal statute for the vexatious litigants, neither the scientific journals nor the press have yet reported the existence of an epidemics of self-immolations in public spaces similar to that which hit Algeria, Tunisia, Greece, or France during the economic crisis (Bouazzoni, 2013). Notwithstanding the importance of cultural and economic differences, this situation may be due to the fact that, in countries which have passed Vexatious Litigant Acts (Stauber, 2009), people identified as vexatious litigants can see their name written on a list. Such measure may symbolically offset the loss of their right to appear before a court. Though they can't file new legal proceedings without prior authorization, vexatious litigants may be endowed with a statute that prevents the denial of their very existence. So, the act demanded by these subjects may be a mere legal nomination, an equivalent to the right to a legal existence. For want of clinical data, we can only advance this hypothesis and not prove it yet.

### Querulousness, fear of breakdown, and masochism

On an even more fundamental level, one last analysis might unite our four previous sets of hypotheses. It is often difficult to determine whether the querulous subjects endeavor to settle the real injustices they suffered or if they primarily confront some perceived *sense* of injustice which points at some more primordial longing. This implies that querulous subjects may use the legal battles they wage in order to overcome some deeply rooted feeling of fear, weakness, or insecurity that originated in early traumatic experiences. The narratives of injustice presented to the judges (and to the police officers, clerks, or deputy mayors) may serve as ego-defenses against what Winnicott (1974) called the fear of breakdown.

When such narratives, supported by symbolic acts (e.g., filing legal proceedings), prove unable to solve their deeper, unconscious issues, the plaintiffs may feel doomed to face an earlier core of injustice suffered. In Lacanian terms, this deeper core of traumatic experience that underlies any subject's conscious actions pertains to the real (Lacan, 1954–1955, p. 122 and p. 342; Lacan, 1956–1957, p. 31; Pommier, 2004). Being directly confronted with the anxiety it generates may cause a shift toward self-aggressive gestures. Then, masochistic tendencies would result from a collapse of the ego-defenses which had, so far, protected the querulous persons against some deeply rooted unconscious trauma.

In fact, some traces of masochism seem to appear in the four clinical cases presented in section How Do Querulous Claimants Voice Their Suicidal Thoughts?: Ms Zuliani's self-sacrificial struggle, M. Doux's suicidal thoughts associated with his misfortunes, M. Gac's two hunger-strikes and his self-presentation as a victim, and M. Maubert's enjoyment of his solitude are some phenomena that may illustrate how querulous persons direct their own aggressive feelings against themselves. Self-immolation may result from the growth of these masochistic seeds. Moreover, the metapsychological motive of masochism apparently allows us to better understand why querulousness may, in some cases, be qualified as a paranoid disorder. Since Bak's groundbreaking clinical paper (Bak, 1946) and Deleuze's philosophical contribution (Deleuze, 1961, 1967), French psychoanalysts have underlined the importance of originary masochism in paranoia. In the times to come, we hope to devote a full-length text to the contributions of Racamier (1966), Chasseguet-Smirel (1966), Chazaud (1966), Rosolato (1969), and Rosenberg (1991), Laplanche (1992; 1995) and Mallet (2008). This would enable to show how their theory of masochism in paranoia sheds light on self-immolation of some querulous individuals. Deleuze's work on masochism (Deleuze, 1961) may also help us clarify how the death drive described by Freud (1920a) may be combined with self-destructive behaviors and produce a certain attraction for deleterious *jouissance* (Toscano, 2009; Vialet-Bine, 2009), which results in suicide.

As a last remark, let us underline that, while masochistic tendencies are liable to turn against the querulous person, some other trends of a more sadistic nature do not completely disappear. They may be directed toward the spectators who supposedly watch the scene of self-immolation. The querulous person who self-immolates willingly inflicts on them a vision of horror. Hence, the great importance of the fact that such gestures happen in public places. Like the chorus in ancient Greek tragedy (Rehm, 2009), the witnesses of the self-immolations are endowed with a role: they have to feel frightened and, later, to attest to the fact that the plaintiff died as a martyr. It is possible that terrorist attacks offer to some vulnerable persons an even clearer way for expressing similar tendencies, both on a sadistic and on a masochistic level. Masochism may be expressed by the sacrifice of the person who offers his life to a religious (or a political) cause. Sadism, on the other hand, seems to be clearly directed toward the victims and the spectators of the scene, a phenomenon which is amplified by the media impact of terrorist attacks.

To sum up this point, it can be stated that the querulous individuals who self-immolate and the self-enlisted authors of terrorist acts share the same tendencies to enhance their ego-defenses (McGregor et al., 2015; AFP/Le Point, 2016). Ironically, at some point, protecting their own narcissism may imply giving in to masochistic and sadistic tendencies, and choosing a violent death. Today, such suicidal behaviors might take different forms and prove more ubiquitous in society than they appear.

## Conclusion

We have endeavored to investigate a specific type of self-aggressive act, namely, the self-immolations by which some querulous plaintiffs try to put an end to their lives in public places. At some point, most of them have actually suffered from injustice. Their loss may be petty, but it may also amount to huge sums of money. Other contexts also exist in which these subjects don't manage to assert their rights to housing or employment benefits. This generates a frustration which turns first into anger and then into despair. Public places such as the tribunals, the town halls, and the employment agencies seem to be locations in which their feelings may be expressed, and sometimes through violent gestures endowed with a strong symbolic value. In certain cases, self-immolation may have an important media impact.

As we have shown, the querulous plaintiffs seem to embody the quest for an object, which Lacan has called the “objet petit a” (“small a object”)—that is, the fantasy-object which is the target of the drive. Their quest appears not to take place in a fantasy world but in the real; it is undertaken under other people's gaze. The querulous individuals seem to be guided by their will to be granted some rights over the object. Their complaints might be an attempt to raise the symbolic other's attention via some representative figure (judge, deputy mayor, police officer, etc.). Unfortunately, their efforts can't meet with success: the object of the drive has no empirical or material item (Vanier, 2009). It can't be appropriated or even grasped.

If they become aware that no appropriate response will be given to them, then the querulous plaintiffs may move into a melancholic phase (Reniers et al., 2011; Lévy and Vanier, 2016). Their complaints hide some need to be listened to, which is crushed by the Other's negative reaction. Self-aggressive gestures might happen when these claimants have no more words to voice their request. Their suicidal behaviors constitute a last attempt to draw the other's attention. In such cases, a reversal seems to take place: the plaintiffs become an object for the other. Aggressive feelings, which couldn't be directed toward their opponents, appear to have turned against themselves. This hypothesis implies that, through the suicidal crisis, querulous individuals return to the other the product of an unconscious message: they offer themselves as a sacrifice. On an even deeper level of meaning, their self-aggressiveness may be a means of expression for some unconscious feeling of injustice, accompanied by a fear of breakdown that has enhanced their ego-defenses as well as some deleterious masochistic trends. The same trends have often been seen at work in paranoid disorders and may cause some subjects to enlist in terrorist organizations and commit suicidal attacks. In other words, self-immolation may be only one specific means of expression for self-aggressive tendencies that can take different forms.

Non-suicidal subjects continuously reschedule the payment of their symbolic debts, whereas the querulous claimants seemingly owe their life to the other and could be willing to give it right now. By committing suicide, they may attest to the fact that language has failed to constitute a safety fence against self-aggressive behaviors. Prevention of these plaintiffs' suicidal gesture involves those people in contact with them (e.g., members of the legal professions, social workers, civil servants, and mental health professionals), which openly shows their will to listen to their voice and support their hope (Commonwealth Ombudsman, 2009). These claimants might be asking for a mere permission to go on struggling rather than paying this with their own life. They need to find a place for open communication and expression of their grievances (Fitzroy Legal Service Inc., 2008).

## Author contributions

BL has conducted the study. BL and FL have analyzed the data. BL and CP have analyzed the literature on the topic. BL has written the first draft of the text. CP and RE have re-read the text and participated to its rewriting.

### Conflict of interest statement

The authors declare that the research was conducted in the absence of any commercial or financial relationships that could be construed as a potential conflict of interest.
